# A Case of Persistent Pasteurella multocida Cellulitis Complicated With Large Endocarditis Vegetation

**DOI:** 10.7759/cureus.68825

**Published:** 2024-09-06

**Authors:** Theresia R Davita, Nathan Giunto, Muhammad Husnain, Ken Wong

**Affiliations:** 1 Internal Medicine, Cleveland Clinic Akron General, Akron, USA; 2 Internal Medicine, Northeast Ohio Medical University, Akron, USA; 3 Infectious Disease, Cleveland Clinic Akron General, Akron, USA

**Keywords:** gram negative bacteremia, gram negative endocarditis, lower extremity cellulitis, mitral endocarditis, pasteurella infection, pasteurella multocida, pasteurella spp, valvular endocarditis

## Abstract

In the realm of infective endocarditis, a distinct and infrequent player emerges - *Pasteurella multocida*, an organism more commonly associated with zoonotic infections, now warranting careful consideration in this unique case report. *P. multocida* is a Gram-negative, facultative anaerobic cocco-bacillus and a common member of the oral bacterial flora of cats and dogs. In humans, it commonly causes skin and wound infections after bites and scratches. Disseminated *P. multocida* infection seeded into the heart valve is very rare and has only been reported in about one case per year worldwide with only 42 cases found in the literature and only five cases reported to have underlying liver cirrhosis as in our case.

This is a case of a 73-year-old female with a past medical history of Child-Pugh B liver cirrhosis secondary to primary biliary cholangitis with portal hypertension, splenomegaly, pancytopenia, severe aortic stenosis, and paroxysmal atrial fibrillation presented to hospital with generalized weakness, fever, and new lower extremity rash 48 hours after last dose of antibiotic. She had recent hospitalization for left lower extremity cellulitis and* P. multocida* bacteremia and received 14 days of high-dose oral amoxicillin-clavulanate with negative blood culture prior to discharge. She occasionally helps her son to feed his cats and dog whenever he travels. She was readmitted and a repeat blood culture showed *P. multocida*. Transthoracic echocardiogram showed a 1.9 cm × 1 cm mobile mass attached to the anterior mitral valve leaflet, which was new compared to the prior study obtained during her first admission. She was not a suitable candidate for valve surgery due to her comorbidities. *P.* *multocida* was found to be susceptible to penicillin, ampicillin, levofloxacin with negative beta lactamase. Her cellulitis, fever, and bacteremia eventually resolved with intravenous antibiotics. She was ultimately discharged with a two-week course of intravenous ceftriaxone, continued with oral levofloxacin to complete six weeks of total treatment, and followed by long-term penicillin suppression.

In this case report, we delve into a rare and intriguing clinical presentation of *P. multocida* endocarditis. Our patient is the second reported case which showed complication of native mitral valve endocarditis even in the setting of bacteremia resolution. This report sheds light on the challenging diagnosis and management of this uncommon yet clinically significant condition, highlighting the importance of vigilant and prompt intervention in cases of infective endocarditis with atypical causative agents.

## Introduction

*Pasteurella multocida*, primarily known for its association with animal-related infections [1], has now become more common in a patient population typically at risk for a spectrum of infections due to their compromised immune status. This clinical scenario has taken an unexpected turn, challenging conventional diagnostic paradigms. This case report offers a unique perspective on the pathogenesis of Pasteurella-associated endocarditis, raising intriguing questions about alternative modes of transmission and underlying risk factors in patients with liver cirrhosis. The most recent literature review shows that only 42 cases have previously been published reporting endocarditis caused by *P. multocida* [2]. Among those cases, only five were found to have cirrhosis as a comorbidity [2-6].

Here, we present a comprehensive exploration of the diagnostic challenges, treatment modalities, and clinical outcomes in this rare and intriguing manifestation of endocarditis in the setting of liver cirrhosis in the absence of a history of animal bite. By unraveling the intricacies of this case, we aim to provide valuable insights that can guide clinicians in managing similar scenarios with greater confidence and precision. This article was previously posted to the Access Microbiology preprint server on April 5, 2024.

## Case presentation

A 73-year-old female presented to the hospital with chief complaint of recurrent fever, chills, generalized weakness, and new lower extremity rash 48 hours after completing six days of IV ampicillin-sulbactam 3 g every 6 hours which transitioned to eight days of twice a day oral amoxicillin-clavulanate 875-125 mg with addition of amoxicillin 500 mg for recent admission due to *P. multocida* bacteremia with lower extremity cellulitis. There was absence of complications during the initial treatment of cellulitis at the previous admission, in particular, thrombophlebitis, subcutaneous abscess formation, and heart murmur. The patient mentioned she lives in her apartment and has no pets. She occasionally feeds her son’s Great Dane dog and cats whenever he travels, but has never noticed any history of animal bites. She was hit by the dog’s tail the morning before the hospital admission and immediately developed a rash.

**Figure 1:**
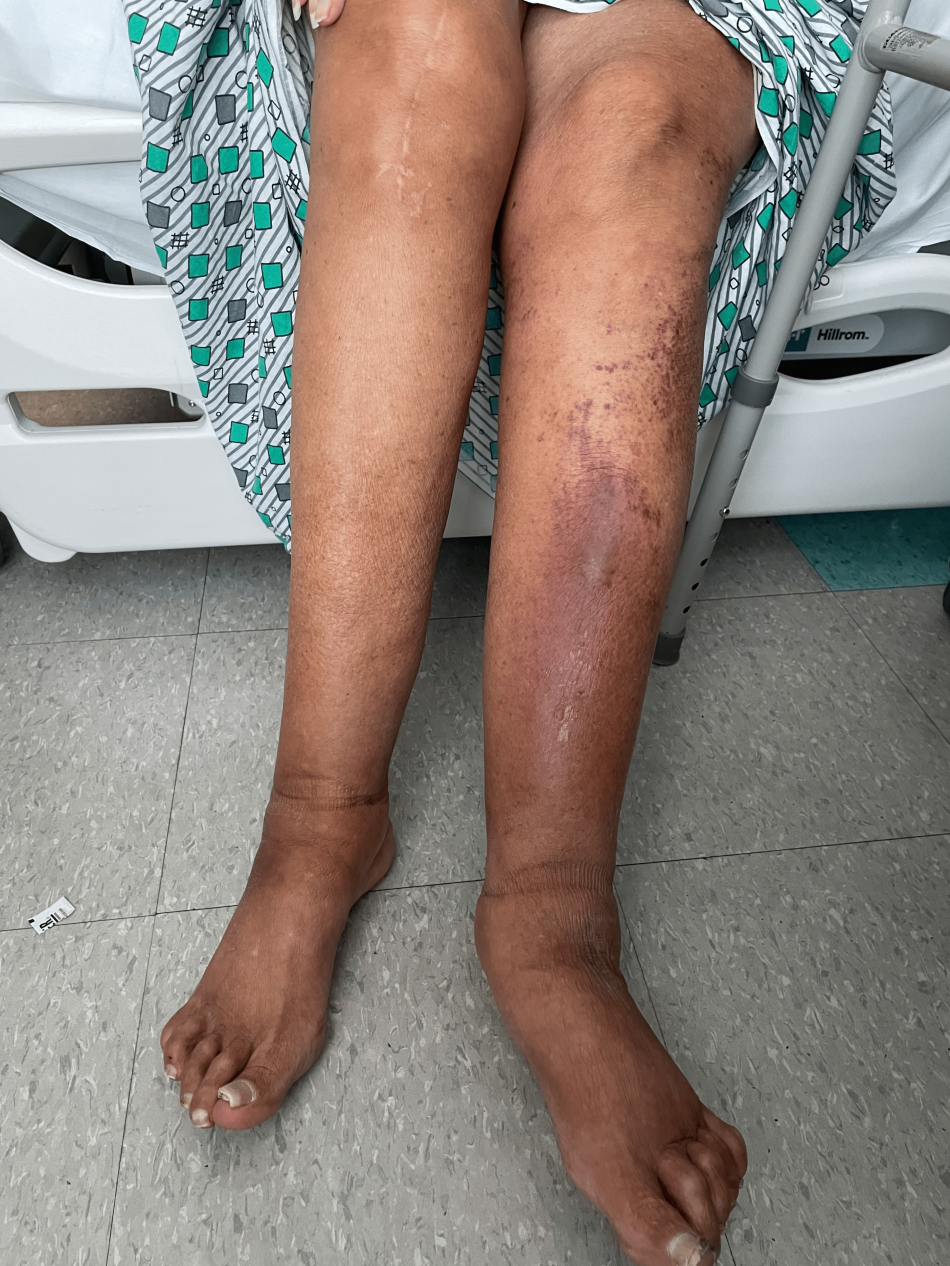
Image of the wound due to the cat scratch on the patient’s left lower extremity. The rash covered the lower two-thirds of the anterior surface of the lower leg with increased swelling and warmth when compared to the right.

The patient had a medical history of Child-Pugh B liver cirrhosis due to primary biliary cholangitis with portal hypertension, splenomegaly, pancytopenia, severe aortic stenosis with obvious crescendo-decrescendo late systolic murmur, and paroxysmal atrial fibrillation. The patient was scratched on her left lower extremity by her son’s cat a week prior to her initial admission which led her to develop cellulitis. Upon her current admission, she had a new purpuric nonblanching rash on the lateral side of her left lower extremity with a tender and warm feeling on her previous cellulitis area (Figure 1).

Diagnostic assessments

**Figure 2:**
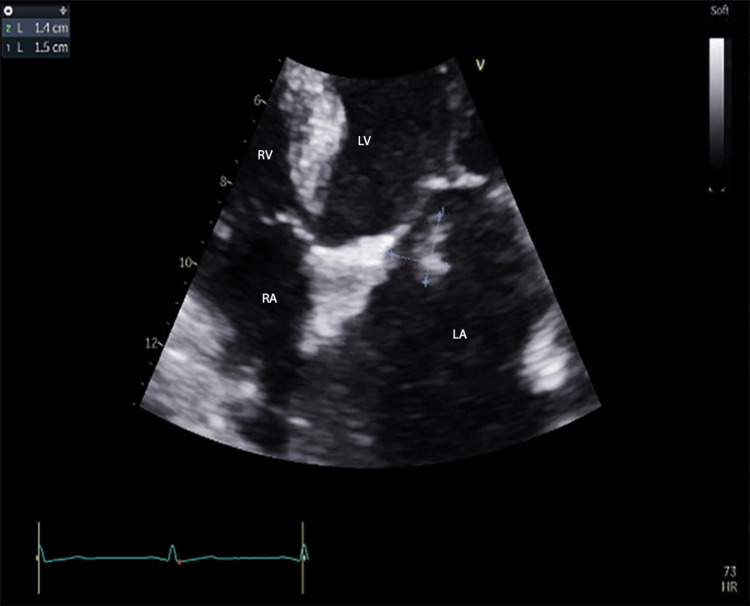
TTE results from the patient’s hospital admission with measurement of the echogenic mass in the atrial aspect of the anterior mitral leaflet. TTE: transthoracic echocardiogram

Blood culture and transthoracic echocardiography findings were negative prior to her initial discharge with amoxicillin-clavulanate 500-125 mg three times daily. During this readmission, a repeat blood culture grew *P. multocida* again, which showed susceptibility to penicillin, ampicillin, and levofloxacin with negative beta-lactamase. A transthoracic echocardiogram was further pursued due to relapse/persistence of bacteremia without antibiotic resistance, which showed a new 1.9 cm × 1 cm mobile echogenic structure attached to the mitral valve leaflet (Figure 2). A transesophageal echocardiography (TEE) was not done because the patient was clinically stable, she just had esophageal varices banding and since not a surgical candidate, TEE will not impact the plan.

Therapeutic interventions

Intravenous piperacillin-tazobactam was initiated during the admission and then narrowed down to ceftriaxone 2 g daily on the third day of hospitalization after the susceptibility result. The patient was not a candidate for valve surgery due to her comorbidities. Her cellulitis and bacteremia eventually resolved. She was discharged to the skilled nursing facility with the plan to receive a 20-day course of intravenous ceftriaxone via an upper extremity peripherally inserted central catheter (PICC) line then continued with oral levofloxacin 750 mg daily to complete six weeks of total treatment followed by long-term penicillin suppression.

Follow-up and outcome

The patient became neutropenic after receiving ceftriaxone for 14 days with a WBC count of 2200 per microliter at the lowest. Thus, the patient was transitioned to levofloxacin early. She has since been discharged home from the nursing facility and is doing well on levofloxacin with improved symptoms, and complete resolution of swelling and redness. The patient is currently still on long-term penicillin V potassium 500 mg twice daily.

## Discussion

General characteristics and transmission of Pasteurella bacteria

*Pasteurella* species are Gram-negative facultative anaerobic bacteria belonging to the *Pasteurellaceae* family. These bacteria are widely distributed in the environment and can be found in the oral and nasopharyngeal flora of various animals, including domestic pets like dogs and cats, as well as livestock. They are responsible for a range of infections in both animals and humans. While *P. multocida* is one of the most clinically significant species within this genus, it is important to understand the general characteristics and transmission routes of *Pasteurella* bacteria before delving into the specifics of *P. multocida* [7].

*Pasteurella* organisms are non-motile, pleomorphic rods that do not form spores. They are oxidase-positive and ferment glucose, producing acid but not gas. These characteristics aid in their identification in the laboratory setting [7]. Among the various *Pasteurella* species, *P. multocida* is particularly notable due to its role in human infections, which are typically associated with animal bites and scratches.

The transmission of *Pasteurella* bacteria to humans primarily occurs through close contact with infected animals, especially bites and scratches. Pasteurella organisms can colonize the oral and respiratory tracts of animals, contaminating their saliva. When these animals bite or scratch humans, the bacteria can be introduced into the skin, leading to localized infections. Common animal reservoirs for Pasteurella include dogs and cats, although other animals, such as rodents and farm animals, can also harbor these bacteria [8, 9].

Moreover, Pasteurella bacteria can be found in the environment, including soil and water, which may serve as indirect sources of infection. Contaminated wounds or punctures, as well as inhalation of aerosolized particles in certain occupational settings (such as farming), can also lead to Pasteurella infections in humans [7, 10].

*Pasteurella multocida*: *A Closer Look*

*P. multocida* is the most clinically significant species within the Pasteurella genus. It is associated with a wide range of clinical manifestations in humans, including skin and soft tissue infections, respiratory tract infections, and, less commonly, systemic infections.

The transmission of *P. multocida* to humans is primarily linked to animal-related exposures. Bites, scratches, and close contact with cats and dogs are the most common routes of transmission [8]. Unlike some other zoonotic bacteria, such as Bartonella sp., *P. multocida* infections can occur in individuals with minimal contact with animals and may even affect those who do not own pets.

Our case highlights a unique instance where *P. multocida* infection seeded a heart valve, leading to endocarditis. Notably, the patient did not report a history of direct animal bites but instead mentioned occasional contact and scratches of her son’s cats and dog. This case underscores the potential for *P. multocida* to cause severe and unusual infections in individuals with limited animal exposure, especially in the presence of underlying comorbidities, as seen in this patient with liver cirrhosis.

In conclusion, comprehending the general characteristics and transmission routes of *Pasteurella* bacteria, particularly *P. multocida*, is crucial for diagnosing and managing infections caused by these organisms. While animal bites and scratches remain the primary modes of transmission, this case emphasizes the need for heightened clinical vigilance, especially in immunocompromised individuals, as Pasteurella infections can present atypically and lead to severe complications.

Case discussion

The liver plays a significant role in the maintenance of the immune system in our body [11]. Disturbances in liver function have been associated with a significant risk of infection. It has been reported that cirrhotic liver was the most common underlying comorbidity present in patients with Pasteurella bacteremia [12]. A cirrhotic liver is associated with the development of cirrhosis-associated immune dysfunction (CAID) which increases vulnerability to risk of infection and progression to systemic infection as it impairs bacteria and endotoxin clearance leading to disseminated bacterial infection including bacteremia and its associated complications [13]. CAID causes continuous immune system cell activation with the production of pro-inflammatory cytokines which affect both adaptive and innate immunity which in combination with portosystemic shunt increases systemic infection and fatality of sepsis up to 30% [14].

Pasteurella endocarditis is a rare complication of Pasteurella infection which has only been reported in 42 cases in literature with 26% fatality [2]. Among those rare cases, only five cases to date reported Pasteurella endocarditis in patients with underlying liver cirrhosis with a 60% mortality risk, more than double the mortality risk compared to patients without underlying cirrhosis (Table 1) [2-6]. All of those cases involved *P. multocida*.

**Table 1:** Patient data from six cases of Pasteurella endocarditis, including the one in this study

Author	Year of Publication	Age	Sex	Outcome	Surgery	Treatment (Duration)	Valve Involved	Native Valve Disease	Animal Encounter	Cirrhosis Etiology
Guerain et al. [6]	1980	62	F	Died	No	None	Aortic	None	Cat contact	Alcoholic liver cirrhosis
Genne et al. [4]	1996	38	F	Cured	No	Penicillin, Ceftriaxone (6 weeks)	Aortic	None	Cat contact	Chronic hepatitis C
Vasquez et al. [5]	1998	65	M	Died	No	Ampicillin-sulbactam (16 hours)	Aortic	Aortic Stenosis	Dog licking	Cryptogenic cirrhosis
Fayad et al. [3]	2003	48	M	Cured	Yes	Cefotaxime+Gentamicin (3 weeks) +valve replacement; Vancomycin+Cefotaxime+Gentamycin (6 weeks)	Aortic	None	Dog contact	Hepatic cirrhosis
Mahmoud et al. [2]	2022	47	M	Died	No	Vancomycin+Cefepime (3 days) Ceftriaxone+Metronidazole (3 days) Vancomycin+Piperacillin-Tazobactam (1 day)	Mitral	None	Cat scratch	Alcoholic liver cirrhosis
Davita et al.	2024	73	F	Cured	No	Piperacillin-Tazobactam (2 days), Ceftriaxone (2 weeks) Levofloxacin (4 weeks); Life-long penicillin	Mitral	Aortic Stenosis	Cat scratch	Primary biliary cholangitis

The rarity of *P. multocida* endocarditis gave little guidance to establish proper management of patients with *P. multocida* endocarditis with underlying cirrhosis. Randall et al. suggested that surgical intervention should be offered to all patients with Pasteurella endocarditis based on a statistical analysis of 16 patients who had excellent outcomes (100% survival) after undergoing surgery [15]. However, the severity of the presenting clinical condition was not taken into account when the author made that statement from the paucity of data available to them at that time. Carter et al. reported that small Pasteurella endocarditis could be treated with six weeks intravenous antibiotic regimen without surgical intervention [16]. It showed that not all endocarditis needs surgical interventions, and surgical intervention indication could be consistent with the current guideline from the Infectious Disease Society of America (IDSA). Among two reported cases of *P. multocida* patients with underlying liver cirrhosis who survived [3, 4], one was treated successfully with valve surgery, and one was with antibiotics only.

Our case report adds to another successful case treated promptly with antibiotics in an early presentation of *P. multocida* endocarditis. This is significant because there is a lack of adequate data on treating endocarditis with *P. multocida*, especially in patients with major comorbidities such as liver cirrhosis, which requires aggressive treatment. Our patient fulfilled IDSA criteria for valve surgery (Table 2), however, surgery was deferred because she was a high risk due to her severe cirrhotic liver with multiple associated complications which include, but not limited to, hypersplenism and pancytopenia. Fortunately, the patient presented early for her second readmission which showed that her persistent infection was further complicated by the development of endocarditis despite negative blood culture conversion and continuously taking proper antibiotic with only 48-hour time elapsed between the last oral antibiotic to reemerging sepsis. This interesting phenomenon apparently was previously seen in the fatal case reported by Mahmoud et al. whose patient also had underlying cirrhosis. In his case, the patient had a continuously growing endocarditis size despite initial clinical improvement and continuous intravenous antibiotics administration [2]. We believe underlying liver cirrhosis and its associated immunocompromised state play a major role in the lack of suppression of Pasteurella endocarditis growth.

**Table 2:** Clinical and echocardiographic features in infective endocarditis that suggest potential need for surgical intervention based on IDSA IDSA: Infectious Disease Society of America

Clinical and Echocardiographic Features That Suggest Potential Need for Surgical Intervention	
Vegetation	
	Persistent vegetation after systemic embolization
	Anterior mitral leaflet vegetation, particularly with size >10 mm
	≥1 Embolic events during the first 2 weeks of antimicrobial therapy
	Increase in vegetation size despite appropriate antimicrobial therapy
Valvular Dysfunction	
	Acute aortic or mitral insufficiency with signs of ventricular failure
	Heart failure unresponsive to medical therapy
Valve Perforation or Rupture	
	Perivalvular extension
	Valvular dehiscence, rupture, or fistula
	New heart block
	Large abscess or extension of abscess despite appropriate antimicrobial therapy

Learning from these two cases, we suggest the need for close monitoring of Pasteurella endocarditis patients with underlying cirrhosis despite initial evidence of clinical improvement. Prolonged monitoring in hospitals and further investigation of deep-seated infections such as infective endocarditis and infected thrombus may be justified if the patient improves but continues to have persistent evidence of systemic infection. Repeat limited echocardiogram within one week of initial study as well as having low threshold for transesophageal echocardiogram can be considered as even though endocarditis is rare, high fatality if detected late with severe presentation should be put into consideration. Early detection with less severe presenting symptoms is the common aspect among three survived cases (including our case) of *P. multocida* endocarditis cases in cirrhotic liver patients.

## Conclusions

In certain compromised patients, *P. multocida* infection can lead to sepsis, and, in rare cases, to life-threatening endocarditis. Early antibiotic treatment is paramount to potentially avoiding valve surgery, or in our case, life-saving as surgery was not an option. We agree with the most commonly suggested antibiotic duration of six weeks antibiotic as the most common treatment chosen among clinicians who managed disseminated Pasteurella endocarditis.

In patients with Pasteurella infection, proper surgical revision is necessary as mentioned in the IDSA guidelines. Antibiotic treatment and patient compliance are necessary components to avoid complications. If reduced compliance is suspected, hospitalization during treatment should be considered. Special consideration should be given to patients with diabetes mellitus, ongoing immunosuppression, or alcohol overconsumption. The need for life-long antibiotics prophylaxis might need to be considered in patients with a history of Pasteurella endocarditis with close contact with domesticated animals such as dogs or cats. Once again, the paucity of data on this rare case prohibited us from creating an evidence-based plan, however, we believed this a reasonable treatment strategy for this potentially fatal case.
